# *Parthenium hysterophorus:* A Probable Source of Anticancer, Antioxidant and Anti-HIV Agents

**DOI:** 10.1155/2013/810734

**Published:** 2013-11-17

**Authors:** Shashank Kumar, Gousia Chashoo, Ajit K. Saxena, Abhay K. Pandey

**Affiliations:** ^1^Department of Biochemistry, University of Allahabad, Allahabad 211002, India; ^2^Cancer Pharmacology Division, Indian Institute of Integrative Medicine, Jammu 180001, India

## Abstract

The present work reports the anticancer, antioxidant, lipo-protective, and anti-HIV activities of phytoconstituents present in *P. hysterophorus* leaf. Dried leaf samples were sequentially extracted with nonpolar and polar solvents. Ethanol fraction showed noticeable cytotoxic activity (81–85%) in SRB assay against MCF-7 and THP-1 cancer cell lines at 100 **μ**g/ml concentration, while lower activity was observed with DU-145 cell line. The same extract exhibited 17–98% growth inhibition of HL-60 cancer cell lines in MTT assay, showing concentration dependent response. Ethanol extract caused 12% reduction in mitochondrial membrane potential and 10% increment in sub G1 population of HL-60 cell lines. Several leaf fractions, namely, ethyl acetate, ethanol, and aqueous fractions exhibited considerable reducing capability at higher concentrations. Most of the extracts demonstrated appreciable (>75%) metal ion chelating and hydroxyl radical scavenging activities at 200 *µ*g/ml. All the extracts except aqueous fraction accounted for about 70–80% inhibition of lipid peroxidation in rat liver homogenate indicating protective response against membrane damage. About 40% inhibition of reverse transcriptase (RT) activity was observed in hexane fraction in anti-HIV assay at 6.0 *µ*g/ml concentration. The study showed that phytochemicals present in *P. hysterophorus* leaf have considerable potential as cytotoxic and antioxidant agents with low to moderate anti-HIV activity.

## 1. Introduction

 Identification of novel therapeutic agents as new drugs for alleviation of the human suffering from cancer and other degenerative diseases is of prime concern [1, 2]. Excessive generation of reactive oxygen species (ROS) may cause oxidative stress, cytotoxicity, and cell death due to structural alterations of cellular molecules [3]. Antioxidants play a significant role to combat oxidative stress and prevent diseases by scavenging free radicals or chelating trace elements and thereby protecting antioxidant defenses [4]. 

Phytoconstituents acting as antioxidants are believed to reduce the risk of cancer [5]. It is generally believed that chemotherapeutic agents in the treatment of cancer cells induce apoptosis. Apoptosis results from the activation of caspases, the cysteine proteases, in response to different cell death stimuli via extrinsic and/or intrinsic pathways [6, 7]. The extrinsic pathway involves the activation of cell surface death receptors (Fas antigen) leading to the activation of caspase 8. On the other hand, intrinsic pathway is triggered due to the release of cytochrome c in the cytosol from mitochondria which results in the formation of a multi-protein complex apoptosome and leads to activation of caspase 3, 6, and 9 [8]. This brings changes in the mitochondrial membrane that ultimately results in an opening of the mitochondrial permeability transition pore (MPT), loss of mitochondrial transmembrane potential (ΔΨ_*m*_), and release of proapoptotic proteins from the intermembrane space into the cytosol [9]. The normal mechanism of cell cycle check point controls are often disrupted during tumor genesis which gives cells the chance to escape from apoptosis and proliferate continuously [10].

Although iron is vital for life, it can be toxic when it is present in excess [11]. About 0.1% of body iron circulates in the plasma as an exchangeable pool, essentially all bound to transferrin. The process of chelation not only facilitates the transport of iron into cells, but also prevents iron-mediated free radical toxicity. The toxic effects of free iron are substantiated by its ability to catalyze via Fenton reaction the generation of damaging reactive free radicals including the hydroxyl radical [12]. Peroxidation of lipids is also mediated by iron with the generation of peroxyl radicals (ROO^•^) which ultimately leads to malondialdehyde (MDA) production. MDA can form adduct with DNA bases, and significantly elevated levels of adducts with guanine have been reported in human breast tissues [13].

About 30.8 million people are infected with HIV/AIDS out of which 95% of them live in the developing countries. The development of virus resistance is a continuing problem [14]. Natural products have been found to inhibit unique enzymes and proteins crucial to the life cycle of HIV [15]. The toxicity of currently available anti-HIV drugs makes it difficult to maintain patient's adherence to antiretroviral therapy [16]. As a consequence, the search for better anti-HIV agents continues, and much attention has been focused on natural sources, particularly plant species.

*Parthenium hysterophorus* L. (Asteraceae), a weed, also known as congress grass is an annual herb. Plant has been used as folk remedy for the treatment of infectious and degenerative diseases [17–19]. In India and many other countries, extracts of *P. hysterophorus* are used as ethnomedicine against inflammatory, skin, neural diseases and female reproductive problems [20, 21]. The leaf extracts have a role in the fertility, fecundity and behavioral response [22]. Some researchers have reported its use in traditional medicine for treatment of wounds, ulcerated sores, fever, anemia, and heart troubles [23]. The review of literature indicates that no systematic study has been conducted regarding biological application of sequentially extracted fractions from *P. hysterophorus* leaves. Present work was therefore undertaken to evaluate the anticancer, antioxidant, lipo-protective, and anti-HIV activities of various *P. hysterophorus* leaf extracts.

## 2. Materials and Methods

### 2.1. Plant Material and Preparation of Extracts

**Figure 1:**
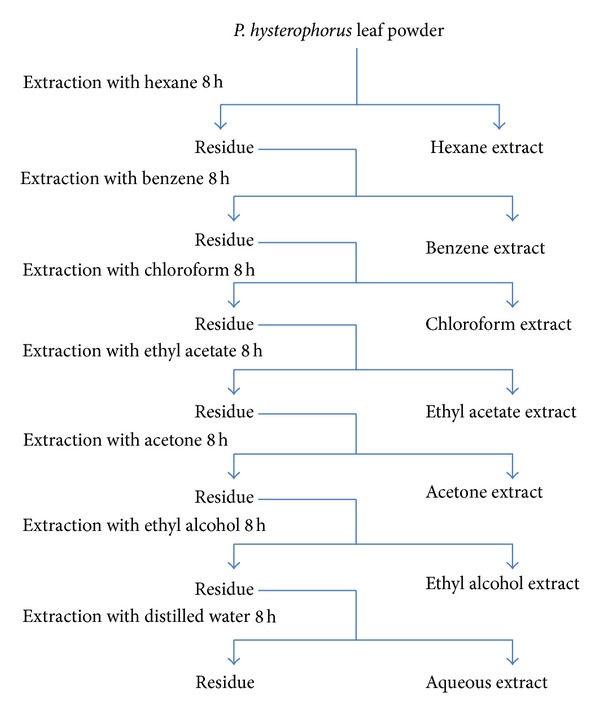
Schematic representation of sequential extraction of *P. hysterophorus* leaf.

The *P. hysterophorus* leaves were collected in May 2010 from the Science Faculty Campus, University of Allahabad, Allahabad, India. The shade-dried leaves were crushed and ground into fine powder with mortar and pestle. Powdered material was sequentially extracted with hexane (HX), benzene (BZ), chloroform (CH), ethyl acetate (EA), acetone (AC), ethyl alcohol (ET), and water (AQ) in Soxhlet apparatus as described earlier [19, 24]. The schematic representation of extraction is shown in Figure 1. The respective extract fractions were centrifuged, filtered, and lyophilized. The dried residues were dissolved in DMSO for determination of anticancer, antioxidant, and anti-HIV activities, while for lipid peroxidation inhibition (LPOI) studies the residues were dissolved in respective solvents.

### 2.2. Cell Lines, Growth Conditions, and Treatment

Human cancer cell lines, namely, breast cancer (MCF-7), leukemia (THP-1), prostate cancer (DU-145) and promyelocytic leukemia (HL-60), were procured from National Center for Cell Sciences, Pune, India. Cell lines were grown and maintained in RPMI-1640 medium, pH 7.4 with 10% FCS, 100 units/mL penicillin, 100 *μ*g/mL streptomycin, and 2 mM glutamine. Cells were grown in CO_2_ incubator (Heraeus, GmbH Germany) at 37°C in the presence of 90% humidity and 5% CO_2_.

### 2.3. Cytotoxic Assay by Sulforhodamine B Dye (SRB Assay)

The *in vitro *cytotoxicity of leaf extracts was determined using sulforhodamine-B (SRB) assay [25]. Cell suspension (100 *μ*L, 10^5^ to 2 × 10^5^ cells per mL depending upon mass doubling time of cells) was grown in a 96-well tissue culture plate and incubated for 24 hours. 100 *μ*L test extract (100 *μ*g/well) was then added to the wells and cells were further incubated for another 48 h. The cell growth was arrested by layering 50 *μ*L of 50% TCA and incubated at 4°C for an hour followed by washing with distilled water and then air dried. SRB (100 *μ*L, 0.4% in 1% acetic acid) was added to each well and plates were incubated at room temperature for 30 min. The unbound SRB dye was washed with 1% acetic acid and then plates were air dried. Tris-HCl buffer (100 *μ*L, 0.01 M, pH 10.4) was added and the absorbance was recorded on ELISA reader at 540 nm. Suitable blanks and positive controls were also included. Each test was done in triplicate. The value reported here is mean ± SD of three experiments.

### 2.4. MTT Assay (Cell Proliferation Inhibition Assay)

*In vitro* cell proliferation assay was done using MTT [3-(4,5-dimethylthiazol-2-yl)-2,5-diphenyltetrazolium bromide] assay [26]. Cell suspension (100 *μ*L) was incubated for 24 h followed by addition of 100 *μ*L extracts (100 *μ*g/well) and further incubated for 72 h. MTT solution (10 *μ*L) was added to each of the 96 wells, and then plates were wrapped with aluminum foil and incubated at 37°C for 4 h leading to the formation of MTT-formazon crystals. Absorbance was measured in ELISA reader at 540 nm. Controls and samples were assayed in triplicate. The results are shown as mean ± SD.

### 2.5. Mitochondrial Membrane Potential (ΔΨ_*m*_) Assay

Detection of mitochondrial permeability transition event provides an early indication of the initiation of cellular apoptosis. This process is typically confined to the collapse of the electrochemical gradient across the mitochondrial membrane as measured by the change in the membrane potential (ΔΨ_*m*_). The loss of the mitochondrial membrane potential is indicative of apoptosis and can be measured after staining with Rhodamine-123 [27]. Exponentially growing HL-60 cells (1 × 10^6^ /mL/well) were treated with ET extract (100 *μ*g/well) for 24 h. Rhodamine-123 (200 nM) was added 1 h before the termination of the experiment. Cells were collected, washed in phosphate buffered saline (PBS), and incubated with propidium iodide (5 *μ*g/mL) for 15 min. The decrease in fluorescence intensity due to the loss of mitochondrial membrane potential was analyzed using flow cytometry in FL-1 channel. Camptothecin was used as positive control.

### 2.6. Cell Cycle Analysis

Nuclear DNA at sub G1 phase in normal and extract treated HL-60 cancer cell lines was estimated by cell cycle analysis using flow cytometer. HL-60 cell lines (2 × 10^6^/mL) were treated with ET extract (100 *μ*g/mL) for 24 h and washed twice with ice-cold PBS, harvested, fixed in cold 70% ethanol in PBS, and stored at −20°C for 30 min. After fixation, the cells were incubated with RNase A (0.1 mg/mL) at 37°C for 30 min, stained with propidium iodide (50 *μ*g/mL) for 30 min on ice in the dark [28], and then measured for nuclear DNA content using BD-LSR flow cytometer (Becton Dickinson, USA) equipped with electronic doublet discrimination capability using blue (488 nm) excitation from argon laser. The fluorescence intensity of sub G1 cell fraction represented the apoptotic cell population.

### 2.7. Metal Ion Chelating Activity

The chelation of ferrous ions by the *P. hyterophorus* leaf extracts was estimated by the method of Dinis et al. [29] as modified by us [30]. Briefly the extracts samples of different concentrations were added to a solution of 2 mmol/L ferric chloride (0.05 mL). The reaction was initiated by the addition of 5 mmol/L ferrozine (0.2 mL), and the mixture was shaken vigorously and left standing at room temperature for 10 min. Absorbance of the solution was then measured spectrophotometrically at 562 nm. The inhibition percentage of ferrozine-Fe^2+^ complex formation was calculated by the formula given below.

(1) $$\begin{array}{c} \text{Metal}\;\text{ion}\;\text{chelating}\;\text{ability}\left(\%\right)=\left[\frac{A_{0}/A_{1}}{A_{0}}\right]\times 100, \end{array}$$

where *A*_0_ is the absorbance of control and *A*_1_ the absorbance in the presence of test sample. 

### 2.8. Hydroxyl Radical Scavenging Activity

Hydroxyl radical scavenging activity (HRSA) was estimated by the method of Klein et al. [31]. Aliquots of extracts (100 *μ*L) were taken in different amounts in test tubes. One milliliter of Fe-EDTA solution (0.13% ferrous ammonium sulfate and 0.26% EDTA), 0.5 mL of 0.018% EDTA, and one mL of 0.85% (v/v) DMSO (in 0.1 M phosphate buffer, pH 7.4) were added to the test tubes, followed by 0.5 mL of 0.22% (w/v) ascorbic acid. The tubes were capped tightly and incubated on a water bath at 85°C for 15 min. After incubation, the test tubes were uncapped and 1 mL of ice-cold TCA (17.5% w/v) was added in each immediately. Three milliliters of Nash reagent (7.5 g of ammonium acetate, 3.0 mL glacial acetic acid, and 2.00 mL acetyl acetone were mixed and made up to 100 mL with distilled water) was added to all the tubes and incubated at room temperature for 15 min. Absorbance was measured at 412 nm. Percentage HRSA was calculated by the following formula:

(2) Hydroxyl  radical  scavenging  activity(%)  =[A0−A1A0]×100,

where *A*_0_ is absorbance of the control and *A*_1_ is that of test samples.

### 2.9. Lipid Peroxidation Inhibition Assay (LPOI Assay)

The method described by Halliwell and Gutteridge [32] was followed to determine the amount of malondialdehyde (MDA) formation with slight modifications [4, 30]. Liver of normal albino Wistar rats was isolated and 10% (w/v) homogenate was prepared in phosphate buffer (0.1 M, pH 7.4 having 0.15 M KCl) with homogenizer (REMI) at 4°C. The homogenate was centrifuged at 800 g for 15 min, and clear cell-free supernatant was used for the study of *in vitro* lipid peroxidation. 100 *μ*L extract solutions (2 *μ*g/*μ*L) were prepared in respective solvents and evaporated to dryness followed by addition of 1 mL potassium chloride (0.15 M) and 0.5 mL of rat liver homogenate. Peroxidation was initiated by adding 100 *μ*L ferric chloride (10 mM). After incubation at 37°C for 30 min, lipid peroxidation was monitored by the formation of thiobarbituric acid reactive substances (TBARS). TBARS were estimated by adding 2 mL of ice-cold HCl (0.25 N) containing 15% trichloroacetic acid (TCA), 0.5% TBA, and 0.5% butylated hydroxytoluene (BHT) to the reaction mixture, followed by heating at 100°C for 60 min. The samples were then cooled and centrifuged, and absorbance of the supernatants was measured at 532 nm. The percent LPOI of extracts was calculated as follows:

(3) $$\begin{array}{c} \left(\%\right)\text{LPOI}=\left[\frac{A_{C}-A_{S}}{A_{C}}\right]\times 100, \end{array}$$

where *A*_*C*_ is absorbance of control and *A*_*S*_ is absorbance of sample solution. 

### 2.10. Reducing Power Assay

Reducing power was determined by the method of Oyaizu [33] with slight modifications [34]. One mL aliquots of extracts (200, 400, 600, 800, and 1000 *μ*g/mL) prepared in DMSO were taken in test tubes. To each test tube, 2.5 mL of phosphate buffer (0.2 M, pH 6.6) and 2.5 mL of 1% potassium ferrocyanide (K_3_Fe (CN)_6_) were added and contents were mixed. Tubes were then incubated at 50°C for 20 min. The reaction was stopped by adding 2.5 mL of 10% TCA solution and then centrifuged at 4000 g for 10 min. One mL of the supernatant was mixed with 1 mL of distilled water and 0.5 mL of ferric chloride solution (0.1% w/v) and kept at room temperature for 2 min. The absorbance was measured at 700 nm. BHT was used as positive control for comparison. All the tests were run in triplicate. Results are reported as mean ± SD. Higher absorbance indicated the higher reducing power.

### 2.11. Anti-HIV Activity

The HIV-RT inhibition assay was performed by using an RT assay kit (Roche) [35]. Briefly, the reaction mixture consists of template/primer complex, dNTPs, and reverse transcriptase (RT) enzyme in the lysis buffer with or without extract/inhibitors. After 1 h incubation at 37°C, the reaction mix was transferred to streptavidin-coated microtitre plate (MTP). The biotin-labeled dNTPs that are incorporated in the template due to activity of RT were bound to streptavidin. The unbound dNTPs were washed using wash buffer and anti-digoxigenin-peroxide (anti-DIG-POD) was added to the MTP. The DIG-labeled dNTPs incorporated in the template were bound to anti-DIG-POD antibody. The unbound anti-DIG-POD was washed and the peroxide substrate (ABTS) was added to the MTP. A colored reaction product was produced during the cleavage of the substrate catalyzed by a peroxide enzyme. The absorbance of the sample was determined at 405 nm using microtiter plate ELISA reader. The resulting color intensity is directly proportional to the actual RT activity. The percentage inhibitory activity of RT inhibitors (extracts) was calculated by comparing to a sample that does not contain an inhibitor using the formula given below:

(4) $$\begin{array}{c} \%\;\text{Inhibition}=100-\left[\frac{A_{\text{WI}}}{A_{\text{WOI}}}\times 100\right], \end{array}$$

where *A*_WI_ is absorbance of control and *A*_WOI_ is absorbance of sample solution.

## 3. Results

### 3.1. Cytotoxic Activity of Extracts by SRB Assay

**Figure 2:**
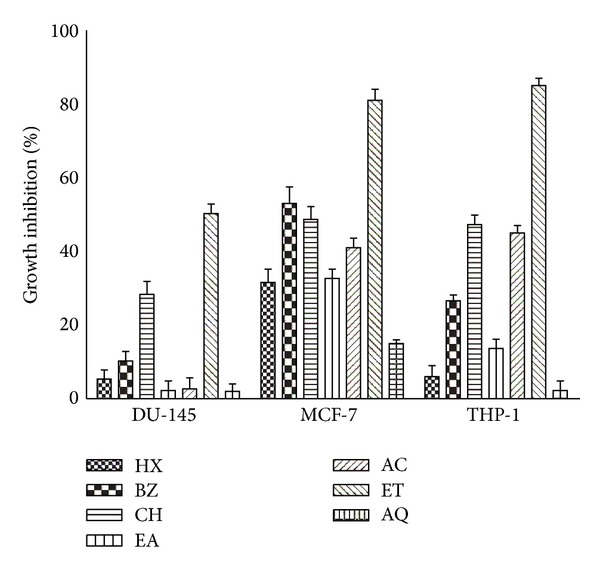
Cytotoxic effect of *P. hysterophorus* leaf extracts against different cancer cell lines using SRB assay. Percentage growth inhibition of DU-145 (prostate), MCF-7 (breast), and THP-1 (leukemia) cancer cell lines was assayed at 100 *μ*g/mL concentration of extracts as described in Section 2. Abbreviations: HX-hexane, BZ-benzene, CH-chloroform, EA-ethyl acetate, AC-acetone, ET-ethanol, and AQ-water. Data represent mean ± SD of three replicates (*P* < 0.05).

The cytotoxicity activity of *P. hysterophorus* leaf extracts was tested against three cancer cell lines, namely DU-145 (prostate), MCF-7 (breast) and THP-1 (leukemia) at the concentration of 100 *μ*g/mL using SRB assay and results are shown in Figure 2. Considerable inhibitory potential among test extracts was observed in ET fraction against MCF-7 (81%) and THP-1 (85%) cell lines. ET fraction also produced about 50% inhibition against DU-145. CH and AC fractions produced moderate (41–50%) cytotoxic activity against MCF-7 and THP-1 cell lines. BZ fraction accounted for 53% cytotoxic activity against MCF-7 cell lines only.

### 3.2. Growth Inhibitory Activity of Extracts in MTT Assay

**Figure 3:**
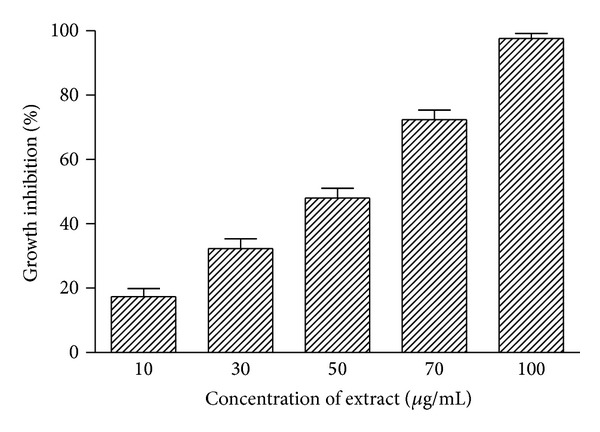
MTT assay of *P. hysterophorus* leaf ET extracts against HL-60 cancer cell line. Percentage growth inhibition of HL-60 (promyelocytic leukemia) cancer cell line was assayed at different concentrations (10–100 *μ*g/mL) of ethanol (ET) extract as described in Section 2. Data represent mean ± SD of three replicates (*P* < 0.05).

The ET fraction exhibiting appreciable cytotoxicity against breast and leukemia cancer cell lines in SRB screening was further assayed for its antitumour potential against HL-60 (promyelocytic leukemia) cell lines at different concentrations of the extract (10, 30, 50, 70, and 100 *μ*g/mL) using MTT assay. An increasing growth inhibitory activity in the range 17–98% was observed with increasing concentration of ET extract showing dose dependent response (Figure 3). At highest test concentration cytotoxic activity was more pronounced as indicated by about 98% cell growth inhibition potential.

### 3.3. Mitochondria Membrane Potential Assay

**Figure 4:**
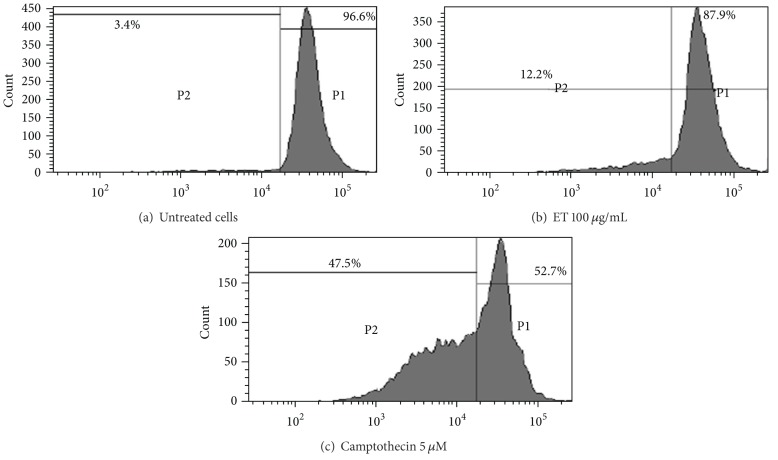
Loss of mitochondrial membrane potential (ΔΨ_*m*_) in* P. hysterophorus* leaf ET extract treated HL-60 cells. (a) Untreated cells (Control), (b) treated cells, and (c) positive control. HL-60 cells (1 × 10^6^/mL/well) were incubated with indicated doses of ethanol (ET) extract and camptothecin for 24 h. Cells were stained with Rhodamine-123 (200 nM) for 1 h and analyzed in FL-1 versus FL-2 channels of flow cytometer as described in materials and methods section. Data are representative of one of three similar experiments.

HL-60 cells when analyzed for mitochondrial membrane potential loss (ΔΨ_*m*_) after 24 h growth in culture employing Rh-123 uptake by flow cytometry revealed that almost all the cells were functionally active with high Rh-123 uptake fluorescence in untreated cells (Figure 4(a)), while the cells exposed to ET fraction for 24 h caused mitochondrial damage resulting in the loss of mitochondrial membrane potential. As evident from the results, the mitochondrial membrane potential loss was found to be 12.2% at a concentration of 100 *μ*g/mL of ET fraction (Figure 4(b)). Camptothecin (5 *μ*M) was used as positive control which under similar conditions showed 47.5% decrease in ΔΨ_*m*_ (Figure 4(c)). 

### 3.4. Cell Cycle Analysis

**Figure 5:**
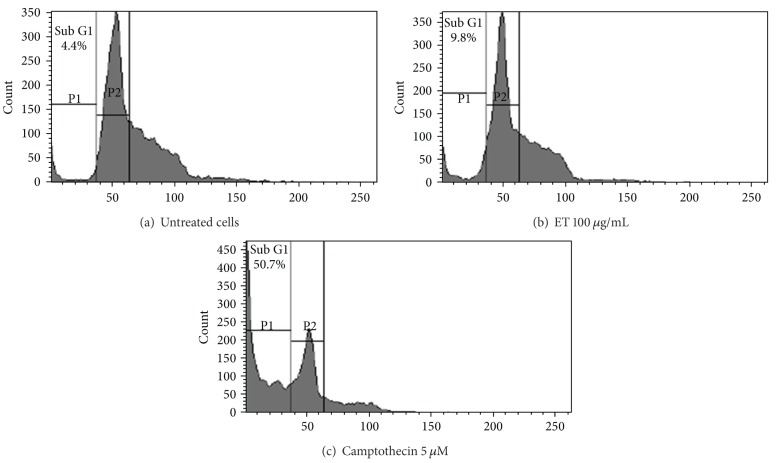
Cell cycle analysis of *P. hysterophorus* leaf ET extract treated HL-60 cells. (a) Untreated cells (control), (b) treated cells, and (c) Positive control. HL-60 cells (2 × 10^6^ cells/mL/well) were exposed to the indicated concentrations of ethanol (ET) extract and camptothecin for 24 h and stained with propidium iodide to determine DNA fluorescence and cell cycle phase distribution as described in materials and methods section. Fraction of cells for sub-G1 population analyzed from FL-2 versus cell counts is shown. Data are representative of one of three similar experiments.

An effective strategy to inhibit tumor growth is deregulated cell cycle progression in cancer cells. Therefore, effect of ET fraction of *P. hysterophorus* leaf on cell cycle progression in HL-60 cells was examined. Cells were treated with test extract at the concentration of 100 *μ*g/mL for 24 h and fluorescence activated cell sorting (FACS) analysis was done. The DNA histogram showed that ET extract increased hypo diploid sub-G1 DNA fraction (<2*n* DNA) (Figure 5(b)). The sub-G1 DNA fraction was 4.4% in untreated cells (Figure 5(a)). However, after treatment with ET extract at 100 *μ*g/mL it increased up to 9.8%, while camptothecin (5 *μ*M) produced 50.7% increase in sub-G1 DNA fraction (Figure 5(c)).

### 3.5. Metal Ion Chelating Activity

**Figure 6:**
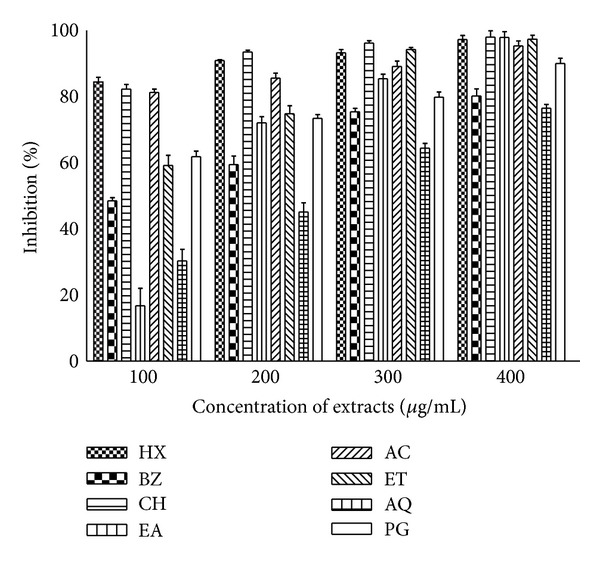
Metal ion chelating ability of *P. hysterophorus* leaf extracts. Phytochemicals present in sample were extracted with hexane (HX), benzene (BZ), chloroform (CH), ethyl acetate (EA), acetone (AC), ethyl alcohol (ET), and water (AQ) as described in methods section. Metal ion chelating activity of extracts and standard antioxidant propyl gallate (PG) was measured at different concentrations and absorbance was recorded at 562 nm. The results are expressed as mean ± SD of three replicates (*P* < 0.05).

*P. hysterophorus* leaf extracts showed an appreciable degree of metal ion chelation ability which is revealed by reduction in formation of red coloured complex. Percent inhibition of colour production as a function of activity of different extracts has been presented in Figure 6. The increasing bar sizes in the figure indicated that formation of the Fe^2+^-ferrozine complex was not complete in the presence of test extracts which demonstrated that most of the extracts have iron chelating ability. The percentage of metal chelating capacity of leaf extracts linearly increased in dose dependent manner from 100 to 400 *μ*g/mL. Even at lowest test concentration about 80% iron was chelated by HX, CH, and AC extracts. The chelating potential of other extracts became more pronounced at higher concentrations. At highest test concentration, HX, CH, EA, AC, and ET fractions showed about 95% metal ion chelating activity. The activity of many extracts was comparatively better than the chelating capacity of PG (propyl gallate) at respective concentrations.

### 3.6. Lipid Peroxidation Inhibition Activity

**Figure 7:**
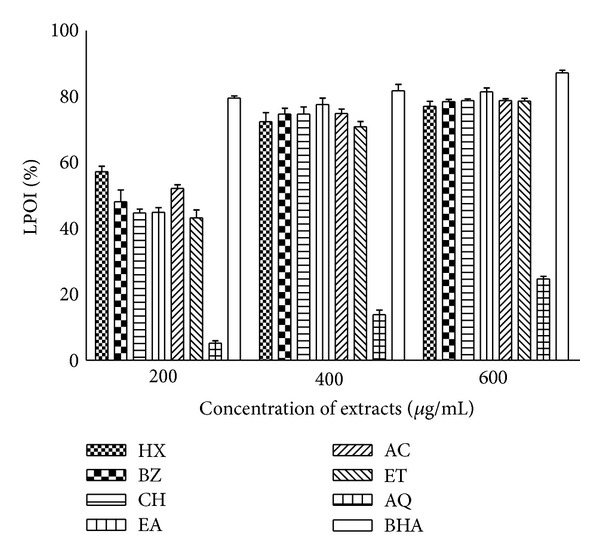
Inhibition of lipid peroxidation (% LPOI) in rat liver tissue by *P. hysterophorus *leaf extracts. Phytochemicals present in leaf were extracted with HX, BZ, CH, EA, AC, ET, AQ, and % LPOI was measured as described in methods section. BHA was used as standard for comparison. The results are expressed as mean ± SD of three replicates with *P* value < 0.05.

The liver homogenate of albino Wistar rats undergoes rapid peroxidation when incubated separately with ferric chloride. The iron induced production of peroxide in turn attacks the biological material. This leads to the formation of MDA (malonodialdehyde) and other aldehydes which form a pink chromogen with TBA showing maximum absorbance at 532 nm [36]. Most of the extracts derived from *P. hysterophorus* leaves showed protection against lipid peroxidation in rat liver homogenate indicating lipo-protective activity in the extracts (Figure 7). The activity was more pronounced at higher concentrations. About 70–77% and 77–81% lipid peroxidation inhibition activities were observed in most of the extracts at 400 and 600 *μ*g/mL concentrations, respectively. AQ fraction was least effective. About 80–87% protection against lipid peroxidation was provided by BHA at all the test concentrations.

### 3.7. Hydroxyl Radical Scavenging Activity

**Figure 8:**
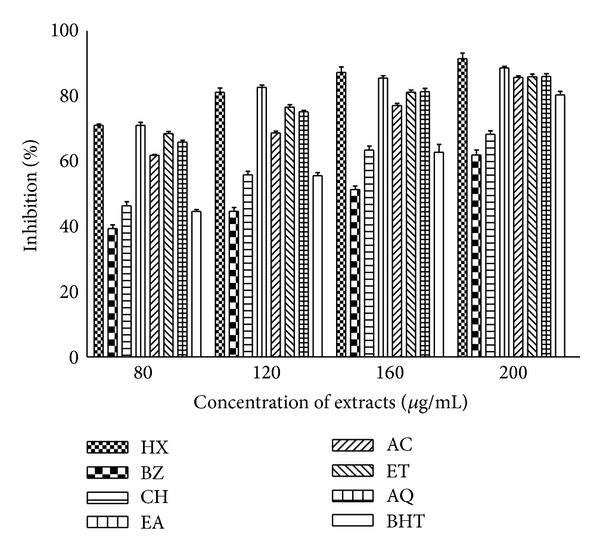
Hydroxy radical scavenging activity (HRSA) of *P. hysterophorus* leaf extracts. Phytochemicals present in leaf were extracted with HX, BZ, CH, EA, AC, ET, and AQ as described in methods section. % HRSA of extracts and standard antioxidant BHT was measured at different concentrations and absorbance was recorded at 412 nm. The data are expressed as mean ± SD of three replicates (*P* < 0.05).

Leaf extracts exhibited radical scavenging potential in dose dependent manner as shown in Figure 8. Noticeable hydroxyl radical scavenging activity (86–92%) was observed in most of the extracts except CH (68%) and BZ (62%) at 200 *μ*g/mL. Most of the polar extracts showed significant free radical quenching potential. Radical scavenging ability of standard antioxidant BHT was comparable to the activity of many extracts.

### 3.8. Reducing Power Assay

**Figure 9:**
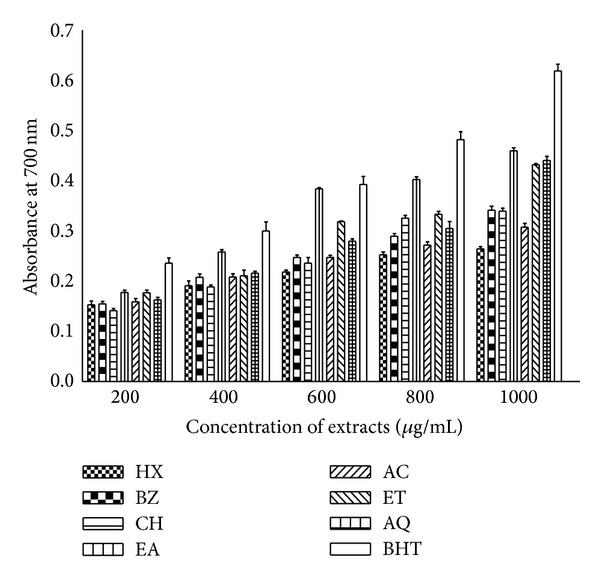
Reducing power assay of *P. hysterophorus* leaf extracts. The extracts were prepared in HX, BZ, CH, EA, AC, ET and AQ as described in Section 2. Reducing power of extracts and standard antioxidant BHT was measured at different concentrations and absorbance was recorded at 700 nm. Data represent mean ± SD of three replicates (*P* < 0.05).

Reducing ability of leaf extracts was measured at different concentrations (200–1000 *μ*g/mL) and results are shown in Figure 9. The colour intensity of reaction mixture indicated the reducing potential. Concentration dependent increase in reducing power was observed. Considerable activity was found in of EA, ET, and AQ fractions at 1000 *μ*g/mL as indicated by absorbance values (0.432–0.460) showing 70–75% activity as compared with positive control (BHT) at the same concentration. However the activity shown by HX, BZ, CH, and AC extracts at same concentration was 42–55% of the activity exhibited by BHT.

### 3.9. Anti-HIV Activity

**Figure 10:**
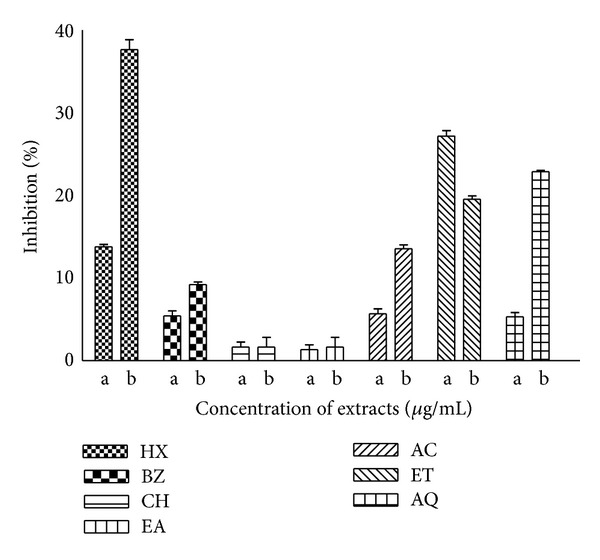
Inhibition of HIV reverse transcriptase (RT) by *P. hysterophorus* leaf extracts. The extracts were prepared in HX, BZ, CH, EA, AC, ET, and AQ as described in Section 2. Anti-HIV-RT activity was measured at two concentrations of extracts, namely, *a* = 0.6 *μ*g/mL and *b* = 6.0 *μ*g/mL. Nevirapine was used as standard anti HIV-RT agent for comparison producing about 99% inhibition of HIV-RT under similar conditions. The data are expressed as mean ± SD of three replicates (*P* < 0.05).

The *P. hysterophorus* extracts were evaluated for antiretroviral activity by targeting HIV reverse transcriptase (RT) enzyme using HIV-RT kit (Roche). Anti-RT activity was measured at two different concentrations (0.6 and 6.0 *μ*g/mL). The extracts showed low inhibition potential (<50%) against RT *in vitro *(Figure 10). Some of the extracts (HX, ET, and AQ) produced modest anti-RT activity (about 23–40%). Nevirapine, the standard anti-HIV drug, showed 99.67% inhibitory activity.

## 4. Discussion

The prevalence of several cancers increases exponentially with age in a human population from the fourth to eighth decade of life. Over 6 million people die due to cancer each year worldwide, being the largest single cause of death in both men and women [37]. According to a study by international agency for research on cancer (IARC), a branch of WHO, there will be approximately 250,000 new cases of breast cancer in India by 2015 [38]. 

About 60% of the anticancer drugs are derived from plant sources, for example, taxol from *Taxus brevifolia* and camptothecin from *Cuscuta reflexa* [39]. Anticancer drug having low side effects, inducing apoptosis and targeting specific cytotoxicity to the cancer cells, is the drug of choice [2, 25]. Keeping this in mind the cytotoxic potential of extracts of *P. hysterophorus *leaves against human prostate (DU-145), breast (MCF-7), and leukemia (THP-1) cancer cell lines were investigated. ET extract was most potent among all the test extracts as it significantly inhibited the cell line proliferation of three different tissues of origin in SRB assay (Figure 2). Cytotoxicity against MCF-7 and THP-1 was more pronounced (80–90% inhibition). ET fraction produced concentration dependent inhibition response and exhibited about 98% cell proliferation inhibition potential against HL-60 cell lines in MTT assay (Figure 3). 

Considering ET fraction of *P. hysterophorus* leaf as a prospective anticancer agent, its apoptotic potential against HL-60 cancer cell lines was also studied using MMP assay and cell cycle analysis. ET extract demonstrated increased hypo diploid sub G1 DNA fraction (<2*n* DNA) of HL-60 cells up to a limited extent at 100 *μ*g/mL test concentration (Figure 5). It was also able to induce partial loss of mitochondrial membrane potential (Figure 4). Loss of mitochondrial membrane potential (ΔΨ_*m*_) is due to the activation of mitochondrial permeability transition pores (PTP) [40]. These two mechanisms produced limited degree of apoptosis and cell cycle arrest under test conditions. However, the observed level of cytotoxicity of extracts in SRB and MTT assays was much higher indicating involvement of some other mechanisms such as inhibition of growth factors leading to cell death [41].

Under stress conditions, availability of “free iron” increases due to release of iron from iron-containing molecules in the body. The process of cellular iron uptake and storage is regulated by iron regulatory proteins; excess iron inactivates iron regulatory proteins (IRP1) [42]. Increased levels of iron in the body enhance risk of a variety of cancer by damaging DNA and causing mutations in Ras genes [43, 44]. Elevated levels of body iron store are involved in the development of hepatocellular carcinoma, colon, and lung cancers [45, 46]. Dysregulation of brain iron homeostasis leads into apoptosis in Alzheimer's disease, which is a hallmark of cancer [36]. 

The transition metal ion, Fe^2+^ possesses the ability to move single electrons by virtue of which it can allow the formation and propagation of many radical reactions, even starting with relatively nonreactive radicals. The main strategy to avoid ROS generation that is associated with redox active metal catalysis involves chelation of the metal ions [47, 48]. Our results have shown that presence of *P. hysterophorus* leaf extracts in reaction mixture led to decline in formation of Fe^2+^-ferrozine complex suggesting chelation of iron by phytochemicals present in this plant. Other reports [2, 18, 30, 49] on chelation of iron by plant extracts also substantiate these findings. It has been reported that chelating agents, which form sigma bonds with a metal, are effective as secondary antioxidants because they reduce the redox potential, thereby stabilizing the oxidized form of the metal ion [49]. The data presented in Figure 6 revealed that most of the extract fractions demonstrated more than 80% iron chelating capacity at 300 *μ*g/mL showing their potential as inhibitors of ROS generation. 

Iron can stimulate lipid peroxidation by the Fenton reaction and also accelerates peroxidation by decomposing lipid hydroperoxides into peroxyl and alkoxyl radicals that can themselves abstract hydrogen and perpetuate the chain reaction of lipid peroxidation [18, 30]. Lipid peroxidation causes damage to unsaturated fatty acids, which results in decreased membrane fluidity and leads to many other pathological events [4, 50]. A correlation study between iron status and atherosclerosis has shown that free or poorly ligated iron can participate in lipid and protein peroxidation [51]. Redox chemistry of iron plays an important role in both the occurrence and the rate of lipid peroxidation. Fe^3+^ reacts with lipid hydroperoxides to form peroxyl radicals that initiate a chain reaction by reacting with other molecules producing MDA, which is usually taken as a marker of lipid peroxidation (LPO) and oxidative stress. Most of the *P. hysterophorus* leaf extracts exhibited considerable lipo-protective efficacy except AQ fraction (Figure 7). It may be inferred that phytoconstituents present in the leaf extracts are responsible for quenching metal ion (Fe), and thereby preventing oxidative damage to lipids leading to protection of liver and other tissues [4]. The study also established strong positive correlation between antilipid peroxidative activity and metal ion chelating activity of the *P. hysterophorus *leaf extracts. Thus, chelation of excess iron by phytoconstituents might be responsible for lowering the oxidative stress as well as risk of iron mediated carcinogenesis [2, 13, 45, 46].

**Figure 11:**
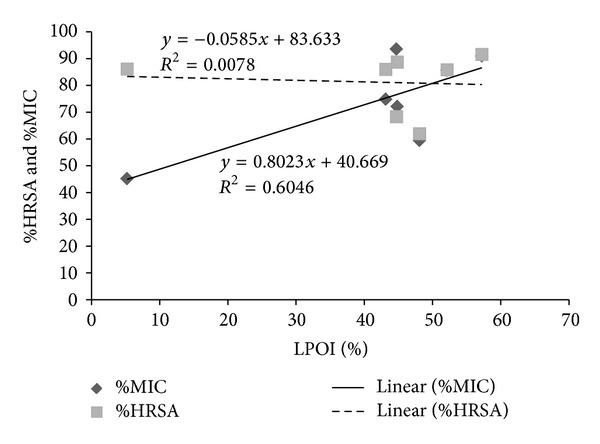
Relationship of % HRSA (hydroxy radical scavenging activity) and % MIC (metal ion chelating ability) of *P. hysterophorus* leaf extracts with % LPOI (lipid peroxidation inhibition) in rat liver homogenate (*P* < 0.05).

A characterized biologic damage by hydroxyl radical is its capacity to stimulate LPO, which occurs when OH radical is generated close to membranes and attacks the fatty acid side chains of the membrane phospholipids [52]. The hydroxyl radical is able to add to double bonds of DNA bases and it abstracts an H-atom from the methyl group of thymine and each of the five carbon atoms of deoxy ribose at a very high rate constant. Permanent modification of genetic material including different malfunctions of cellular process by ROS represents the first step involved in carcinogenesis [53]. Our results displayed that most of the fractions have significant potential (>86%) to scavenge hydroxyl radical (Figure 8). Similar studies have been performed and reported on the protection from hydroxyl radical by other parts of the test plant [18]. Hence, *P. hysterophorus *extracts might be useful to minimize the adverse effects of the hydroxyl radicals. A weak negative correlation was found between % HRSA and % LPOI in rat liver homogenate (Figure 11). It might be inferred that *P. hysterophorus *extracts acting as antioxidants have capacity to combat oxidative damage because of their iron binding and hydroxyl radical scavenging capacities. The latter action also provides protection against cancer initiation. 

The reducing power of a compound is related to its electron transfer ability, and may therefore serve as a significant indicator of its potential antioxidant activity. Fe^3+^ to Fe^2+^ transformation is taken as a measure of reductive ability [49, 54]. It was observed that various extracts derived from *P. hysterophorus* leaves possessed noticeable reducing power in dose dependent manner (Figure 9) as indicated by higher absorbance values which is further substantiated by the reports on other plants [34, 55]. The reducing power implies hydrogen ion donating potential of the *P hysterophorus* leaf extracts. 

Lower anti-HIV-RT activity produced by *P. hysterophorus* leaf extracts indicates that phytoconstituents present in the crude extracts do have antiviral potential at lower test concentration. Since numerous chemical moieties (with or without activity) are present in crude extract, it might be possible that isolation and purification of the active ingredients from potential fractions (HX, ET, AQ) and their bioactivity testing in the future may provide further enhancement in anti-HIV activity [15].

Study demonstrated that various fractions of *P. hysterophorus* leaf have anticancer and antioxidant properties. They are powerful chelators of metal ions and free radical scavengers and could therefore prevent ROS mediated lipid and DNA damage. It may also help in reducing the possibility of hydroxyl radical and metal ion mediated cancer initiation.

## 5. Conclusion

The present study revealed that the phytochemicals present in various *P. hysterophorus* leaf extracts especially in ET fraction possess cytotoxic potential against human cancer cell lines. In addition, *P. hysterophorus* leaf extracts have the capability to combat oxidative damage because of their iron binding, hydroxyl radical scavenging, and lipo-protective activities along with moderate anti-HIV activities.

## References

[1] Newman DJ, Cragg GM, Snader KM (2003). Natural products as sources of new drugs over the period 1981–2002. *Journal of Natural Products*.

[2] Mishra A, Sharma AK, Kumar S, Saxena AK, Pandey AK (2013). *Bauhinia variegata* leaf extracts exhibit considerable antibacterial, antioxidant and anticancer activities. *BioMed Research International*.

[3] Yoshikawa T, Toyokuni S, Yamamoto Y, Naito Y (2000). Free radicals in chemistry, biology and medicine. *OICA International*.

[4] Kumar S, pandey AK (2012). Antioxidant, lipo-protective and antibacterial activities of phytoconstituents present in *Solanum xanthocarpum* root. *International Review of Biophysical Chemistry*.

[5] Kumar S, Pandey AK Chemistry and biological activities of flavonoids: an overview. *The Scientific World Journal*.

[6] Elmore S (2007). Apoptosis: a review of programmed cell death. *Toxicologic Pathology*.

[7] Nicholson DW (1999). Caspase structure, proteolytic substrates, and function during apoptotic cell death. *Cell Death and Differentiation*.

[8] Spee B, Jonkers MDB, Arends B, Rutteman GR, Rothuizen J, Penning LC (2006). Specific down-regulation of XIAP with RNA interference enhances the sensitivity of canine tumor cell-lines to TRAIL and doxorubicin. *Molecular Cancer*.

[9] Saelens X, Festjens N, Vande Walle L, Van Gurp M, Van Loo G, Vandenabeele P (2004). Toxic proteins released from mitochondria in cell death. *Oncogene*.

[10] Mukherjee A, Hazra S, Dutta S (2011). Antitumor efficacy and apoptotic activity of substituted chloroalkyl 1H-benz[de]isoquinoline-1,3-diones: a new class of potential antineoplastic agents. *Investigational New Drugs*.

[11] Lee DW, Andersen JK, Kaur D (2006). Iron dysregulation and neurodegeneration: the molecular connection. *Molecular Interventions*.

[12] Kakhlon O, Cabantchik ZI (2002). The labile iron pool: characterization, measurement, and participation in cellular processes. *Free Radical Biology and Medicine*.

[13] Wang Z, Rossman TG, Chang T (1996). The carcinogenicity of arsenic. *Toxicology of Metals*.

[14] Pomerantz RJ, Horn DL (2003). Twenty years of therapy for HIV-1 infection. *Nature Medicine*.

[15] Cos P, Maes L, Vanden Berghe D, Hermans N, Pieters L, Vlietinck A (2004). Plant substances as anti-HIV agents selected according to their putative mechanism of action. *Journal of Natural Products*.

[16] Siliciano JD, Kajdas J, Finzi D (2003). Long-term follow-up studies confirm the stability of the latent reservoir for HIV-1 in resting CD4^+^ T cells. *Nature Medicine*.

[17] Knox J, Jaggi D, Paul MS (2011). Population dynamics of *Parthenium hysterophorus* (Asteraceae) and its biological suppression through *Cassia occidentalis* (Caesalpiniaceae). *Turkish Journal of Botany*.

[18] Kumar S, Mishra A, Pandey AK (2013). Antioxidant mediated protective effect of *Parthenium hysterophorus* against oxidative damage using *in vitro* models. *BMC Complementary and Alternative Medicine*.

[19] Pandey AK (2007). Anti-staphylococcal activity of a pan-tropical aggressive and obnoxious weed *Parthenium hysterophorus*: an in vitro study. *National Academy Science Letters*.

[20] Recio MC, Giner RM, Uriburu L (2000). In vivo activity of pseudoguaianolide sesquiterpene lactones in acute and chronic inflammation. *Life Sciences*.

[21] Ramos A, Rivero R, Visozo A, Piloto J, García A (2002). Parthenin, a sesquiterpene lactone of *Parthenium hysterophorus* L. is a high toxicity clastogen. *Mutation Research*.

[22] Kumar S, Singh AP, Nair G (2011). Impact of *Parthenium hysterophorus* leaf extracts on the fecundity, fertility and behavioural response of *Aedes aegypti* L. *Parasitology Research*.

[23] Das B, Venkataiah B, Kashinatham A (1999). (+)-Syringaresinol from *Parthenium hysterophorus*. *Fitoterapia*.

[24] Mishra AK, Mishra A, Kehri HK, Sharma B, Pandey AK (2009). Inhibitory activity of Indian spice plant *Cinnamomum zeylanicum* extracts against *Alternaria solani* and *Curvularia lunata*, the pathogenic dematiaceous moulds. *Annals of Clinical Microbiology and Antimicrobials*.

[25] Skehan P, Storeng R, Scudiero D (1990). New colorimetric cytotoxicity assay for anticancer-drug screening. *Journal of the National Cancer Institute*.

[26] Tian GH, Meng JL, Xu YH (2003). Study on polysaccharides extraction and determination from wild and growing *Polystictus versicolor* fruit bodies. *Journal of Hanzhong Teacher's College*.

[27] Desagher S, Osen-Sand A, Nichols A (1999). Bid-induced conformational change of Bax is responsible for mitochondrial cytochrome c release during apoptosis. *Journal of Cell Biology*.

[28] Waxman DJ, Schwartz PS (2003). Harnessing apoptosis for improved anticancer gene therapy. *Cancer Research*.

[29] Dinis TCP, Madeira VMC, Almeida LM (1994). Action of phenolic derivatives (acetaminophen, salicylate, and 5-aminosalicylate) as inhibitors of membrane lipid peroxidation and as peroxyl radical scavengers. *Archives of Biochemistry and Biophysics*.

[30] Kumar S, Sharma UK, Sharma AK, Pandey AK (2012). Protective efficacy of *Solanum xanthocarpum* root extracts against free radical damage: phytochemical analysis and antioxidant effect. *Cellular and Molecular Biology*.

[31] Klein SM, Cohen G, Cederbaum AI (1981). Production of formaldehyde during metabolism of dimethyl sulfoxide by hydroxyl radical generating systems. *Biochemistry*.

[32] Halliwell B, Gutteridge JMC (1989). Protection against lipid peroxidation. *Free Radicals in Biology and Medicine*.

[33] Oyaizu M (1986). Studies on products of browning reactions: antioxidative activities of products of browning reaction prepared from glucosamine. *Japanese Journal of Nutrition*.

[34] Pandey AK, Mishra AK, Mishra A, Kumar S, Chandra A (2010). Therapeutic potential of *C. zeylanicum* extracts: an antifungal and antioxidant perspective. *International Journal of Biological and Medical Research*.

[35] Reverse Transcriptase Assay

[36] Bush AI, Curtain CC (2008). Twenty years of metallo-neurobiology: where to now?. *European Biophysics Journal*.

[37] Kaufmann SH, Earnshaw WC (2000). Induction of apoptosis by cancer chemotherapy. *Experimental Cell Research*.

[38] http://rokocancer.org/aboutcancer.

[39] Verma M, Singh SK, Bhushan S (2008). *In vitro* cytotoxic potential of *Polyalthia longifolia* on human cancer cell lines and induction of apoptosis through mitochondrial-dependent pathway in HL-60 cells. *Chemico-Biological Interactions*.

[40] Wang Z, Wang S, Dai Y, Grant S (2002). Bryostatin 1 increases 1-*β*-D-arabinofuranosylcytosine-induced cytochrome c release and apoptosis in human leukemia cells ectopically expressing Bcl-xL. *Journal of Pharmacology and Experimental Therapeutics*.

[41] Verma KS, Asima S, rajesh N, purohit R, singh S, Himata M (2012). In vitro cytotoxicity of *Emblica officinalis* against different human cancer cell lines. *Asian Journal of Pharmaceutical and Clinical Research*.

[42] Deck KM, Vasanthakumar A, Anderson SA (2009). Evidence that phosphorylation of iron regulatory protein 1 at serine 138 destabilizes the [4Fe-4S] cluster in cytosolic aconitase by enhancing 4Fe-3Fe cycling. *Journal of Biological Chemistry*.

[43] Siah CW, Trinder D, Olynyk JK (2005). Iron overload. *Clinica Chimica Acta*.

[44] Vachtenheim J (1997). Occurrence of ras mutations in human lung cancer. *Neoplasma*.

[45] Kowdley KV (2004). Iron, hemochromatosis, and hepatocellular carcinoma. *Gastroenterology*.

[46] Valko M, Morris H, Mazúr M, Rapta P, Bilton RF (2001). Oxygen free radical generating mechanisms in the colon: do the semiquinones of vitamin K play a role in the aetiology of colon cancer?. *Biochimica et Biophysica Acta*.

[47] Mishra A, Kumar S, Bhargava A, Sharma B, Pandey AK (2011). Studies on *in vitro* antioxidant and antistaphylococcal activities of some important medicinal plants. *Cellular and Molecular Biology*.

[48] Kumar S, Gupta A, Pandey AK (2013). *Calotropis procera* root extract has the capability to combat free radical mediated damage. *ISRN Pharmacology*.

[49] Mishra A, Kumar S, Pandey AK Scientific validation of the medicinal efficacy of *Tinospora cordifolia*. *The Scientific World Journal*.

[50] Olabinri BM, Odedire OO, Olaleye MT, Adekunle AS, Ehigie LO, Olabinri PF (2010). In vitro evaluation of hydroxyl and nitric oxide radical scavenging activites of artemether. *Research Journal of Biological Sciences*.

[51] Stadler N, Lindner RA, Davies MJ (2004). Direct detection and quantification of transition metal ions in human atherosclerotic plaques: evidence for the presence of elevated levels of iron and copper. *Arteriosclerosis, Thrombosis, and Vascular Biology*.

[52] Halliwell B (1991). Reactive oxygen species in living systems: source, biochemistry, and role in human disease. *American Journal of Medicine*.

[53] Dizdaroglu M, Jaruga P, Birincioglu M, Rodriguez H (2002). Free radical-induced damage to DNA: mechanisms and measurement. *Free Radical Biology and Medicine*.

[54] Kumar S, Pandey AK (2013). Phenolic content, reducing power and membrane protective activities of *Solanum xanthocarpum* root extracts. *Vegetos*.

[55] Pandey AK, Mishra AK, Mishra A (2012). Antifungal and antioxidative potential of oil and extracts derived from leaves of indian spice plant *Cinnamomum tamala*. *Cellular and Molecular Biology*.

